# Loss of neurovirulence is associated with reduction of cerebral capillary sequestration during acute *Babesia bovis* infection

**DOI:** 10.1186/1756-3305-6-181

**Published:** 2013-06-18

**Authors:** Kerry S Sondgeroth, Terry F McElwain, Andrew J Allen, Annie V Chen, Audrey OT Lau

**Affiliations:** 1 Department of Veterinary Microbiology and Pathology, College of Veterinary Medicine, Washington State University, Pullman, WA 99164-7040, USA; 2 Paul G. Allen School for Global Animal Health, College of Veterinary Medicine, Washington State University, Pullman, WA 99164-7040, USA; 3 Department of Veterinary Clinical Sciences, College of Veterinary Medicine, Washington State University, Pullman, WA 991674-7040, USA

**Keywords:** Babesia bovis, Babesiosis, Cytoadherence, Sequestration, Attenuation, Neurovirulence

## Abstract

**Background:**

Severe neurological signs that develop during acute infection by virulent strains of *Babesia bovis* are associated with sequestration of infected erythrocytes in cerebral capillaries. Serial passage of virulent strains in cattle results in attenuated derivatives that do not cause neurologic disease. We evaluated whether serial passage also results in a loss of cerebral capillary sequestration by examining brain biopsies during acute disease and at necropsy.

**Findings:**

Cerebral biopsies of spleen intact calves inoculated intravenously with a virulent or attenuated strain pair of *B. bovis* were evaluated for capillary sequestration at the onset of babesiosis and during severe disease. In calves infected with the virulent strain, there was a significant increase in sequestration between the first and second biopsy timepoint. The attenuated strain was still capable of sequestration, but at a reduced level, and did not change significantly between the first and second biopsy. Necropsy examination confirmed the second biopsy results and demonstrated that sequestration identified at necropsy reflects pathologic changes occurring in live animals.

**Conclusions:**

Loss of neurovirulence after serial *in vivo* passage of the highly virulent T2Bo strain of *B. bovis* in splenectomized animals is associated with a significant reduction of cerebral capillary sequestration. Previous genomic analysis of this and two other strain pairs suggests that this observation could be related to genomic complexity, particularly of the *ves* gene family, rather than consistent gene specific differences. Additional experiments will examine whether differential gene expression of *ves* genes is also associated with reduced cerebral sequestration and neurovirulence in attenuated strains.

## Findings

## Background

*Babesia bovis* is a tick-borne apicomplexan parasite that infects cattle in tropical and sub-tropical regions worldwide. Neurological signs may accompany fever and anemia during acute infection by many virulent strains [1]. Neurovirulence is associated with infected erythrocytes cytoadhering to endothelial cells, which subsequently leads to sequestration within cerebral capillaries [2-4]. Attenuated strains of *B. bovis* incapable of inducing neurovirulence are derived *in vivo* through rapid serial passage of parental virulent strains, and are currently used as live vaccines in endemic regions [5]. Loss of neurovirulence associated-attenuation is hypothesized to be due to loss of endothelial cell cytoadherence and sequestration. However, the absence of sequestration has not been proven experimentally, particularly in live animals during acute infection. The mechanism of cytoadherence is postulated to involve endothelial cell binding of infected erythrocytes through interaction of the large protein gene family, Variant Erythrocyte Surface Antigen (VESA)-1, with the endothelial cell surface, similar to the mechanism of sequestration and neurovirulence in falciparum malaria involving the PfEMP1 protein family [6,7].

In this study, we tested whether the loss of neurovirulence in animals infected with the attenuated strain is due to the absence of cerebral capillary sequestration. Temporal evaluation of capillary sequestration was performed *in vivo* by cerebral biopsies during acute infection, and in tissues taken at necropsy.

## Methods

### Strains

The T2Bo virulent strain of *B. bovis* and an attenuated derivative strain obtained after 29 passages in splenectomized calves was utilized. The virulent parental (T2Bo_vir) and attenuated derivative strains (T2Bo_att) have been previously validated to reflect their designated phenotypes of neurovirulence and non-neurovirulence, respectively [8].

### Animals

The study utilized spleen intact Holstein cattle >12months of age. Calves were initially obtained at 3–6 months of age from a Washington State dairy, quarantined at the Washington State University Animal Resource Unit, and given health checks at the Veterinary Teaching Hospital (College of Veterinary Medicine, Washington State University). All cattle were free of previous *B. bovis* infection as determined by sourcing from the United States (which is declared free of bovine babesiosis by the World Organization for Animal Health) and by confirmation through the absence of serum antibody and parasites by cELISA (VMRD, Pullman, WA) and qPCR, respectively, as previously described [9]. All animal procedures were approved by the Animal Care and Use Committee, #ASAF 03322–002.

### Brain biopsy

Two groups of spleen intact cattle (n = 2 for each group) were intravenously inoculated with blood stabilates containing 2 × 10^7^ infected erythrocytes (iRBC) of T2Bo_vir or T2Bo_att strain. Rectal temperature, hematocrit, and parasitemia were monitored daily. One day after the onset of fever (>103°F), animals were anesthetized, placed in sternal recumbency, and a brain biopsy performed at the Veterinary Teaching Hospital (Washington State University, Pullman WA). Cerebral cortical tissue was obtained using a 14 gauge side-cutting disposable brain biopsy needle (Ad-Tech, Racine WI). Multiple 2 mm × 1 cm pieces of biopsy tissue were fixed in 10% buffered neutral formalin for subsequent microscopic evaluation. Following the first biopsy, each animal was recovered from anesthesia and clinical signs monitored. The biopsy process was repeated within 2–3 days when clinical parameters indicated severe clinical disease. All animals were euthanized after the second biopsy while under anesthesia to evaluate capillary sequestration levels after the cessation of blood flow.

Post-mortem brain and tissue samples were collected in duplicate. One sample was placed in 10% buffered neutral formalin for fixation, and the second sample was frozen in liquid nitrogen for DNA extraction and qPCR analysis.

### Microscopic evaluation

Formalin fixed tissues were paraffin embedded, cut into 5 micron sections, placed on glass slides and stained with Giemsa (Washington Animal Disease Diagnostic Laboratory). Sequestration of *B. bovis* iRBC in tissue capillaries was evaluated histologically by counting a minimum of 100 erythrocytes (RBC) in longitudinal sections of capillaries. Manual counts were performed in duplicate at 63X magnification, and the average percentage of infected erythrocytes (iRBC) in capillaries was determined for each tissue using the formula: (iRBC/total RBC) × 100. All tissue sections were examined by the same individual without knowledge of which strain the animal was inoculated with.

### Quantitative PCR on collected tissues

Genomic DNA was extracted from frozen tissues (brain, lung, heart, kidney, liver, spleen, and skin) stored at −80°C. Tissues were thawed, homogenized and lysed using MP Biomedicals lysing matrix A as per manufacturer’s recommendation (MP Biomedicals, Solon OH). Following the first centrifugation step, the supernatant was incubated at 56°C with proteinase K from the Qiagen Blood and Tissue DNA Extraction Kit (Qiagen, Valencia, CA), and extracted following the manufacturer’s recommendation (Qiagen, Valencia, CA). Equivalent starting amounts of tissues (0.1 g) were processed in triplicate for each animal from each group. Quantitative PCR (qPCR) was performed using a previously published protocol targeting the merozoite surface antigen-1 gene (*msa-*1), with the exception of the 5′ primer (5′-GAT GCG TTT GCA CAT GCT AAG-3′). A positive control containing 10 ng of T2Bo gDNA, and negative control of naïve cow gDNA were included [9,10].

### Statistical analysis

Comparative analysis of sequestration at different biopsy time points, and tissue parasitemia, were analyzed using a 2-way ANOVA with repeated measures or 2-way ANOVA, respectively, with each followed by Bonferroni’s multiple comparison tests. A one-tailed student’s t-test was used for parasitemia comparison in blood (Graphpad Prism v. 5.0a).

## Results

**Figure 1:**
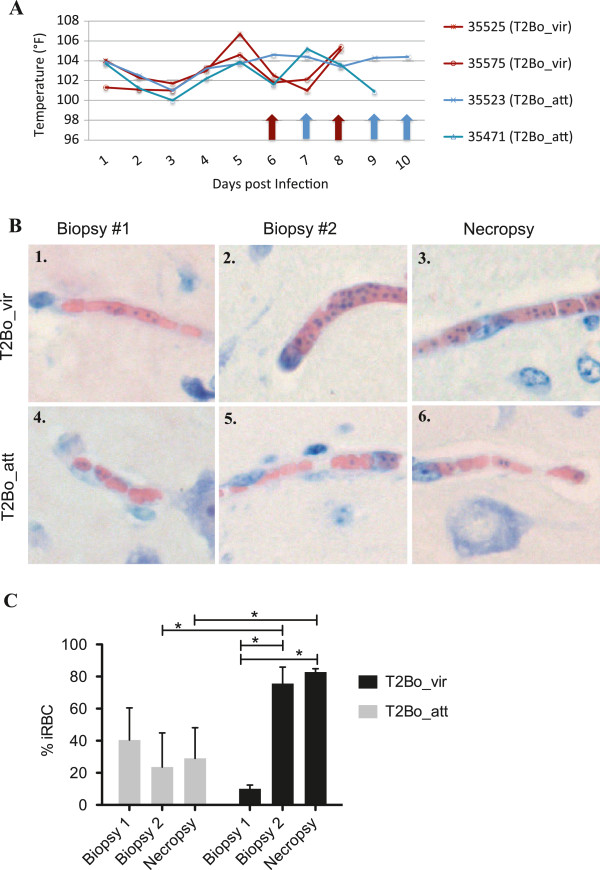
**Sequential evaluation of cerebral capillary sequestration during acute babesiosis. (A)** The timing of biopsy correlated with changes in rectal temperature during infection. Animals infected with T2Bo_vir are in red, and animals infected with T2Bo_att are in blue. The red arrows at days 6 and 8 indicate the first and second biopsy for T2Bo_vir infected animals. The blue arrows at days 7 and 9–10 indicate the first and second biopsy, respectively, for T2Bo_att infected animals. **(B)** Representative photomicrographs of brain biopsies of either the T2Bo_vir or T2Bo_att infected group. First biopsy (panels 1 and 4) was taken one day following the onset of fever. Second biopsy (panels 2 and 5) was taken 2–3 days following the first biopsy. Panels 3 and 6 represent samples taken within one hour of death. **(C)** Levels of sequestration observed in brain biopsies. There is a significant difference in sequestration between first and second biopsies (p < 0.0001), as well as first biopsy and necropsy (p < 0.0001) for the T2Bo_vir group. A significant difference between the T2Bo_vir and T2Bo_att groups for second biopsy and necropsy (p < 0.05) was also observed.

A cerebral cortical biopsy was obtained from all infected animals at the onset of clinical signs as determined by the first day of fever (103°F) (Figure 1A). All biopsied animals received at least one dose of flunixin meglumine (1.1 mg/kg) to control localized inflammation and pain, which resulted in a slight decrease in body temperature in three of four animals. To avoid any confounding effect from this treatment, the second biopsies were obtained in both groups when severe clinical disease with fever was present (Figure 1A).

Cerebral sequestration of iRBC was observed in both T2Bo_vir and T2Bo_att infected animals throughout the experiment (Figure 1B)*.* There was no significant difference in sequestration levels between T2Bo_vir and T2Bo_att infected animals at the onset of clinical signs (biopsy 1). However, the sequestration level in the T2Bo_vir infected animals increased significantly between the first and second biopsy (mean of 10.1% and 75.6% iRBC, respectively; p < 0.0001), as well as between the first biopsy and necropsy (mean of 10.1% and 82.8% iRBC, respectively; p < 0.0001) (Figure 1C). In contrast, there was no significant elevation in sequestration level in T2Bo_att infected animals between the first and second biopsy or first biopsy and necropsy (mean of 40.42%, 23.6%, and 29.0% respectively). The capillary sequestration level between T2Bo_vir and T2Bo_att infected animals at the time of the second biopsy and necropsy were also significantly different (Figure 1C, mean of 75.6% vs 23.6% (second biopsy), and 82.8% vs 29.0% (necropsy), respectively; p < 0.05).

The only statistically significant differences in the level of parasite numbers between the T2Bo_vir and T2Bo_att groups were in the cerebrum and kidney, with significantly higher number of parasites found in the T2Bo_vir group (Table 1). This observation could be due to the tissue-tropism of the virulent strain and corroborates previous reports of sequestration of virulent strains of *B. bovis* in brain and renal capillaries [2].

**Table 1:** Parasite numbers in tissue (copies/g) of T2Bo_vir and T2Bo_att infected animals

**Tissue**	**T2Bo_vir [SEM]**	**T2Bo_att [SEM]**
Cerebrum	8.92E + 05^**a**^ [3.14E + 05]	2.06E + 05 [6.80E + 04]
Lung	6.91E + 04 [6.10E + 03]	2.07E + 04 [6.27E + 03]
Heart	2.70E + 05 [6.49E + 04]	5.21E + 04 [1.44E + 04]
Liver	4.41E + 04 [1.23E + 04]	1.30E + 04 [1.47E + 03]
Kidney	1.26E + 06^**a**^ [3.31E + 05]	1.09E + 05 [1.98E + 04]
Spleen	6.44E + 05 [3.16E + 05]	9.15E + 04 [4.11E + 04]
Skin	0.00E + 00 [0.00E + 00]	0.00E + 00 [0.00E + 00]

^a^Significant difference between the two groups in the cerebrum (p < .05) and kidney (p < .0001).

While *in vivo* brain biopsies provide a unique analysis during acute disease, the challenges of performing these biopsies in large animals limits the total number of animals that can be examined in a study. Nonetheless, even with a small group size, our findings that the overall sequestration was lower in T2Bo_att infected animals than those infected with the parental virulent strain are statistically significant. Factors that may impact these results include variation in the initial or circulating parasite load, and differences in innate or adaptive immune response to parental and attenuated strains. Since the number of infected erythrocytes used in the inoculum for both groups was the same, the experiment was terminated prior to development of a protective immune response, and the time course of the experiment varied by only two days, the differential tissue sequestration levels observed are likely due to the nature of the inocula.

The presence of sustained sequestration with no signs of neurovirulence leads us to conclude that cytoadherence and sequestration, while associated with neurovirulence, may not be solely responsible. One explanation of the findings is that the attenuated strain may have reverted to neurovirulence once introduced back into a spleen-intact animal, a phenomenon reported experimentally in *B. bovis*[5,11] and in *Plasmodium* spp. [12-14]. However, in the field this is uncommon as attenuated strains of *B. bovis* have been used for extended periods as field vaccines without reversion to virulence [1]. The lack of neurovirulence in animals infected with T2Bo_att could be due to repertoire differences of parasite-derived proteins involved with cytoadhesion, such as the VESA1, which may then affect the binding of iRBC with endothelial cell receptors [15,16]. Comparative genome analysis of T2Bo_vir and T2Bo_att strains reveals genomic diversity reduction, including changes in the *ves* gene repertoire [8]. This finding supports the suggestion that genomic differences may contribute to phenotypic variability. Alternatively, a threshold level of iRBC cytoadherence within cerebral capillaries may be necessary for clinically expressed neurovirulence.

## Conclusion

We hypothesized that the lack of neurovirulence in T2Bo_att infected animals is associated with an absence of cytoadherence and sequestration in cerebral capillaries. Our results demonstrate that attenuation with loss of neurovirulence does result in a significant reduction, but not total elimination of sequestration. Ongoing experiments will analyze transcriptomic expression profiles between the two members of this strain pair, and evaluate additional strain pairs in order to better understand the mechanisms of attenuation*.*

## Competing interests

The authors declare that they have no competing interests.

## Authors’ contribution

KSS: performed the study, collected and analyzed blood and tissue samples, prepared the manuscript. TFM: assisted with study design, data analysis and interpretation, writing and manuscript revision. AJA: prepared animals before each brain biopsy, assisted during each surgery, monitored animals post surgery, and manuscript revision. ACA: performed the brain biopsy surgery, and manuscript revision. AOTL: assisted with study design, data analysis and interpretation, writing and manuscript revision. All authors read and approved the final manuscript.

## References

[B1] Callow LL Dalgliesh RJ De Vos AJ Development of effective living vaccines against bovine babesiosis–the longest field trial? Int J Parasitol 1997 27 7 747 767 10.1016/S0020-7519(97)00034-9 9279577

[B2] Everitt JI Shadduck JA Steinkamp C Clabaugh G Experimental Babesia bovis infection in Holstein calves Vet Pathol 1986 23 5 556 562 3776013 10.1177/030098588602300503

[B3] Nevils MA Figueroa JV Turk JR Canto GJ Le V Ellersieck MR Carson CA Cloned lines of Babesia bovis differ in their ability to induce cerebral babesiosis in cattle Parasitol Res 2000 86 6 437 443 10.1007/s004360050691 10894468

[B4] MacPherson GG Warrell MJ White NJ Looareesuwan S Warrell DA Human cerebral malaria: A quantitative ultrastructural analysis of parasitized erythrocyte sequestration The Am J Path 1985 119 3 385 401 PMC1888001 3893148

[B5] Callow LL Mellors LT McGregor W Reduction in virulence of Babesia bovis due to rapid passage in splenectomized cattle Int J Parasitol 1979 9 4 333 338 10.1016/0020-7519(79)90083-3 489240

[B6] Avril M Tripathi AK Brazier AJ Andisi C Janes JH Soma VL Sullivan DJ Jr Bull PC Stins MF Smith JD A restricted subset of var genes mediates adherence of Plasmodium falciparum-infected erythrocytes to brain endothelial cells Proc Natl Acad Sci USA 2012 109 26 E1782 E1790 10.1073/pnas.1120534109 22619321 PMC3387091

[B7] Aikawa M Pongponratn E Tegoshi T Nakamura K Nagatake T Cochrane A Ozaki LS A study on the pathogenesis of human cerebral malaria and cerebral babesiosis Mem Inst Oswaldo Cruz 1992 87 Suppl 3 297 301 1343706 10.1590/s0074-02761992000700051

[B8] Lau AO Kalyanaraman A Echaide I Palmer GH Bock R Pedroni MJ Rameshkumar M Ferreira MB Fletcher TI McElwain TF Attenuation of virulence in an apicomplexan hemoparasite results in reduced genome diversity at the population level BMC Genomics 2011 12 410 10.1186/1471-2164-12-410 21838895 PMC3166950

[B9] Howell JM Ueti MW Palmer GH Scoles GA Knowles DP Persistently infected calves as reservoirs for acquisition and transovarial transmission of Babesia bovis by Rhipicephalus (Boophilus) microplus J Clin Microbiol 2007 45 10 3155 3159 10.1128/JCM.00766-07 17687016 PMC2045367

[B10] Bastos RG Ueti MW Guerrero FD Knowles DP Scoles GA Silencing of a putative immunophilin gene in the cattle tick Rhipicephalus (Boophilus) microplus increases the infection rate of Babesia bovis in larval progeny Parasit Vectors 2009 2 1 57 10.1186/1756-3305-2-57 19930572 PMC2785768

[B11] Timms P Stewart NP De Vos AJ Study of virulence and vector transmission of Babesia bovis by use of cloned parasite lines Infect Immun 1990 58 7 2171 2176 2365457 10.1128/iai.58.7.2171-2176.1990 PMC258793

[B12] David PH Hommel M Miller LH Udeinya IJ Oligino LD Parasite sequestration in Plasmodium falciparum malaria: spleen and antibody modulation of cytoadherence of infected erythrocytes Proc Natl Acad Sci USA 1983 80 16 5075 5079 10.1073/pnas.80.16.5075 6348780 PMC384191

[B13] Barnwell JW Howard RJ Miller LH Altered expression of Plasmodium knowlesi variant antigen on the erythrocyte membrane in splenectomized rhesus monkeys J Immunol 1982 128 1 224 226 6172478

[B14] Barnwell JW Howard RJ Coon HG Miller LH Splenic requirement for antigenic variation and expression of the variant antigen on the erythrocyte membrane in cloned Plasmodium knowlesi malaria Infect Immun 1983 40 3 985 994 6189787 10.1128/iai.40.3.985-994.1983 PMC348148

[B15] O’Connor RM Allred DR Selection of Babesia bovis-infected erythrocytes for adhesion to endothelial cells coselects for altered variant erythrocyte surface antigen isoforms J Immunol 2000 164 4 2037 2045 10657656 10.4049/jimmunol.164.4.2037

[B16] Allred DR Al-Khedery B Antigenic variation and cytoadhesion in Babesia bovis and Plasmodium falciparum: different logics achieve the same goal Mol Biochem Parasitol 2004 134 1 27 35 10.1016/j.molbiopara.2003.09.012 14747140

